# Preparation of Sequencing RNA Libraries through Chemical Cross-linking Coupled to Affinity Purification (cCLAP) in *Saccharomyces cerevisiae*

**DOI:** 10.21769/BioProtoc.3029

**Published:** 2018-10-05

**Authors:** Congwei Wang, Julie Weidner, Anne Spang

**Affiliations:** Growth & Development, Biozentrum, University of Basel, Basel, Switzerland

**Keywords:** RNA-Seq, *In vivo* cross-linking, Biotin-streptavidin interaction, RNP, RNP purification, *Saccharomyces cerevisiae*

## Abstract

Ribonucleoprotein particles (mRNPs) are complexes consisting of mRNAs and RNA-binding proteins (RBPs) which control mRNA transcription localization, turnover, and translation. Some mRNAs within the mRNPs have been shown to undergo degradation or storage. Those transcripts can lack general mRNA elements, like the poly(A) tail or 5’ cap structure, which prevent their identification through the application of widely-used approaches like oligo(dT) purification. Here, we describe a modified cross-linking affinity purification protocol (cCLAP) based on existing cross-linking and immunoprecipitation (CLIP) methods to isolate mRNAs which could be deadenylated, decapped and/or partially degraded in mRNPs, opening the possibility to detect different types of non-coding RNAs (ncRNAs). Once isolated, the RNAs are subjected to adapter ligation and subsequently proceeded to Next-generation sequencing (NGS). Due to the fast and efficient cross-linking and quenching steps, this protocol is also suitable for transiently induced mRNP granules. Examples include processing bodies (PBs) or stress granules (SGs) triggered by extrinsic stressors. Its reproducibility and broad applications make this protocol a useful and powerful tool to study the RNA compositions of specific RNPs.

## Background

Characterization of transcripts within the mRNPs is crucial in understanding cellular transcriptional and post-transcriptional processes. Isolation of RNAs from mRNP particles by cross-linking and immunoprecipitation followed by RNA-Seq has become a popular approach to identify the mRNA targets (Tagwerker *et al.*, 2006; Hafner *et al.*, 2010; Kishore *et al.*, 2011). UV and photoactivatable ribonucleoside-enhanced techniques are widely used cross-linking methods in CLIP, however, it is not suitable for liquid cultures such as those used to grow yeast. For these studies, a centrifugation step is required to harvest cells, thus potentially introducing stress which can alter stress-related studies. Additionally, the homogeneity of UV exposure on layered yeast cells cannot be controlled and accurately monitored. In the following protocol, we apply chemical cross-linking with formaldehyde. Formaldehyde can be added directly to liquid cultures, which provides an efficient, quenchable and homogenous cross-linking process. Furthermore, cross-linking with formaldehyde can be reversed by heat, which removes the remaining peptides or residues attached on RNAs, thus ensuring an unbiased cDNA generation by reverse transcription. We used an HBH-tag (His6-biotinylation sequence-His6) for the pulldown because this tag is uniquely well-suited for crosslinking under denaturing condition approaches (Tagwerker *et al.*, 2006, Weidner *et al.*, 2014).

The RNA targets of a number of RBPs, particularly those involved in mRNA turnover, are difficult to identify, since poly(A) tail or 5’ cap structure can be absent. Therefore, the commonly used oligo(dT) purification method generates an incomplete and biased library of mRNAs bound by those RBPs. Compared with the oligo(dT)-based methods, we adapted and improved an affinity purification protocol allowing us to globally isolate the transcripts sequestered by specific mRNP complexes. In summary, this chemical cross-linking coupled to affinity purification (cCLAP) protocol allows unbiased isolation of RNAs present within yeast mRNPs for RNA-Seq analysis.

## Materials and Reagents

1. Pipette tips: Filter tips low retention (SARSTEDT, catalog number: 70.1130.215; 70.760.216; 70.760.219; 70.762.216)
2. Sterile Falcon tubes, 15 ml (SARSTEDT, catalog number: 62.554.002)
3. Sterile Falcon tubes, 50 ml (SARSTEDT, catalog number: 62.547.254)
4. Protein LoBind Tubes, 1.5 ml (Eppendorf, catalog number: 0030108116)
5. DNA LoBind Tubes, 1.5 ml (Eppendorf, catalog number: 0030108051)
6. Safe-Lock microcentrifuge tubes, 2.0 ml (Eppendorf, catalog number: 0030120094)
7. PCR tubes, 0.2 ml (SARSTEDT, catalog number: 72.991.002)
8. Centrifuge bottles (Thermo Fisher Scientific, Nagelgene™, catalog number: 3120-1000)
9. Glass beads, acid-washed 425-600 μm (Sigma-Aldrich, catalog number: G8772)
10. Glass beads, acid-washed 212-300 μm (Sigma-Aldrich, catalog number: G1277)
11. Screw Cap Micro Tube, 2 ml (SARSTEDT, catalog number: 72.693.005)
12. GeneRuler™ Low Range DNA Ladder (Thermo Fisher Scientific, Thermo Scientific™, catalog number: SM1192)
13. Saran wrap
14. Carbon Steel Surgical Scalpel Blade (Swann-Morton, catalog number: 0203)
15. Yeast Extract (BD, Bacto™, catalog number: 212720)
16. Peptone (BD, Bacto™, catalog number: 211830)
17. Agar (BD, Bacto™, catalog number: 214010)
18. D(+)-Glucose 1-hydrate (AppliChem, catalog number: A1349)
19. Calcium chloride dihydrate (Sigma-Aldrich, catalog number: C5080)
20. Liquid nitrogen
21. Formaldehyde solution (Sigma-Aldrich, catalog number: 252549)
22. Tris base (Carl Roth, catalog number: 4855)
23. Hydrochloric acid 37% (HCl) (Carl Roth, catalog number: X942)
24. Glycine (Carl Roth, catalog number: 3908)
25. Ethylenediaminetetraacetic acid (EDTA) (Carl Roth, catalog number: 8043)
26. Ethanol BioUltra (Sigma-Aldrich, catalog number: 51976)
27. [γ-^32^P-ATP]Adenosine 5'-triphosphate (ATP) (HARTMANN ANALYTIC, catalog number: SRP-301)
28. di-Sodium hydrogen phosphate dodecahydrate (Carl Roth, catalog number: T106)
29. Sodium dihydrogen phosphate monohydrate (Merck, catalog number: 106346)
30. Magnesium chloride hexahydrate (MgCl_2_) (Sigma-Aldrich, catalog number: M0250)
31. Nonidet™ P 40 Substitute (NP-40) (Sigma-Aldrich, catalog number: 74385)
32. cOmplete™, Mini, EDTA-free Proteinase Inhibitor Cocktail (Roche Diagnostics, catalog number: 11836170001)
33. Sodium deoxycholate (Sigma-Aldrich, catalog number: 30970)
34. Sodium dodecyl sulfate (SDS) Pellets (Carl Roth, catalog number: 8029)
35. Sodium chloride (Carl Roth, catalog number: 3957)
36. Guanidine hydrochloride (GuHCl) (Carl Roth, catalog number: 0037)
37. TWEEN^®^ 20 (Sigma-Aldrich, catalog number: 93773)
38. Streptavidin Agarose (Thermo Fisher Scientific, Thermo Scientific™, catalog number: 20353)
39. RNase T1 (Thermo Fisher Scientific, Thermo Scientific™, catalog number: EN0541)
40. T4 Polynucleotide Kinase (PNK) (New England Biolabs, catalog number: M0201)
41. Alkaline Phosphatase, Calf Intestinal (CIP) (New England Biolabs, catalog number: M0290)
42. TBE buffer (10x) powder (AppliChem, catalog number: A4348)
43. T4 RNA Ligase 2, truncated (New England Biolabs, catalog number: M0242)
44. T4 RNA Ligase Reaction Buffer (New England Biolabs, catalog number: B0216L)
45. T4 RNA Ligase (Thermo Fisher Scientific, Thermo Scientific™, catalog number: EL0021)
46. NEBuffer™ 3 (New England Biolabs, catalog number: B7203)
47. Taq DNA Polymerase (Roche Diagnostics, catalog number: 11146173001)
48. Adenosine 5'-Triphosphate (ATP) (New England Biolabs, catalog number: P0756)
49. Proteinase K (Roche Diagnostics, catalog number: 03115836001)
50. Phenol-chloroform-isoamyl alcohol mixture (PCI) (Sigma-Aldrich, catalog number: 77619)
51. RiboMinus™ Transcriptome Isolation Kit, yeast (Thermo Fisher Scientific, Invitrogen™, catalog number: K155003)
52. SuperScript™ III Reverse Transcriptase (Thermo Fisher Scientific, Invitrogen™, catalog number: 18080093)
53. Oligo (dT) primer (Promega, catalog number: C1101)
54. Random Primers (Promega, catalog number: C118A)
55. Dimethyl sulfoxide (DMSO) (New England Biolabs, catalog number: B0515)
56. Acrylamide/Bis-acrylamide (Sigma-Aldrich, catalog number: A2917)
57. N,N,N’,N’-tetramethylethylenediamine (TEMED) (Thermo Fisher Scientific, Invitrogen™, catalog number: 15524010)
58. Ammonium peroxydisulphate (APS) (Carl Roth, catalog number: 9592)
59. Urea (AppliChem, catalog number: A1049)
60. GlycoBlue™ Coprecipitant (Thermo Fisher Scientific, Invitrogen™, catalog number: AM9515)
61. 1,4-Dithio-DL-Threitol (DTT) (AppliChem, catalog number: A1101)
62. UltraPure™ Agarose (Thermo Fisher Scientific, Invitrogen™, catalog number: 16500)
63. RNasin^®^ Ribonuclease Inhibitors (Promega, catalog number: N2615)
64. RNA Gel Loading Dye (2x) (Thermo Fisher Scientific, Thermo Scientific™, catalog number: R0641)
65. RedSafe™ Nucleic Acid Staining Solution (Bulldog Bio, catalog number: 21141)
66. NucleoSpin^®^ Gel and PCR Clean-up kit (MACHEREY-NAGEL, catalog number: 740609)
67. Nuclease-Free Water (Thermo Fisher Scientific, Invitrogen™, catalog number: AM9937)
68. Illumina 3’ DNA adapter (RA3) (Microsynth, PAGE-purified)
69. Illumina 5’ RNA adapter (RA5) (Microsynth, PAGE-purified)
70. 19nt RNA size marker (Microsynth, PAGE-purified)
71. RT primer (RA3_RT) ACCTTAAGAGCCCACGGTTCC (Microsynth, PAGE-purified)
72. Illumina RP1 primer (Microsynth, PAGE-purified)
73. Illumina Indexing Primer (RPI1) (Microsynth, PAGE-purified)
74. Illumina Indexing Primer (RPI2) (Microsynth, PAGE-purified)
75. Illumina Indexing Primer (RPI3) (Microsynth, PAGE-purified)
76. Illumina Indexing Primer (RPI4) (Microsynth, PAGE-purified)
77. Deoxynucleotide Solution Mix (dNTPs) (New England Biolabs, catalog number: N0447)
78. Yeast Extract-Peptone-Dextrose (YPD) agar (see Recipes)
79. Yeast Extract-Peptone-Dextrose (YPD) medium (see Recipes)
80. RIPA buffer (see Recipes)
81. Incubation buffer (see Recipes)
82. Wash buffer (see Recipes)
83. PNK buffer (see Recipes)
84. 2x proteinase K buffer (see Recipes)
85. Urea polyacrylamide gel (see Recipes)
86. Tris-HCl buffer (see Recipes)

## Equipment

1. Pipettes (PIPETMAN Classic™) (Gilson, models: P2, P10, P20, P200 and P1000)
2. Orbital shaker (Infors, model: Multitron Standard)
3. Erlenmeyer flasks (DWK Life Sciences, Duran^®^, catalog number: 21 990 27; 21 216 32; 21 216 36; 21 216 44)
4. Nalgene™ Polycarbonate culture flasks (Thermo Fisher Scientific, Thermo Scientific™, catalog number: 4105-2800)
5. Balance (Sartorius, model: AZ612)
6. Balance (Sartorius, model: MA160)
7. Incubator (Memmert, model: ICP500)
8. UV/Visible spectrometer (GE Healthcare, Amersham Biosciences, model: Ultrospec 3100 pro)
9. FastPrep^®^ Cell Disrupter (Thermo Fisher Scientific, Savant, model: FP120)
10. Thermomixer (Eppendorf, model: ThermoMixer^®^ R or equivalent)
11. Ultracentrifuge (Thermo Fisher Scientific, Thermo Scientific™, model: Sorvall™ RC 6 Plus)
12. Cooling centrifuge (Eppendorf, model: 5810 R or equivalent)
13. Cooling microcentrifuge (Eppendorf, model: 5417 R or equivalent)
14. Microcentrifuge (Eppendorf, model: 5415 D or equivalent)
15. Electrophoresis power supply (Bio-Rad Laboratories, model: PowerPac™ Universal Power Supply)
16. Vortex mixer (Scientific Industries, model: Vortex-Genie 2)
17. Phosphorimager (GE healthcare, model: Typhoon FLA 7000)
18. Phosphor screen (Fujifilm, model: FUJICHROME MS)
19. Phosphor screen cassette (Fujifilm, model: BAS series)
20. Thermal Cycler (Bio-Rad Laboratories, model: T100™)
21. Gel imager (ProteinSimple, model: AlphaImager HP)
22. Bioanalyzer (Agilent Technologies, model: 2100)

## Procedure

**Figure 1:**
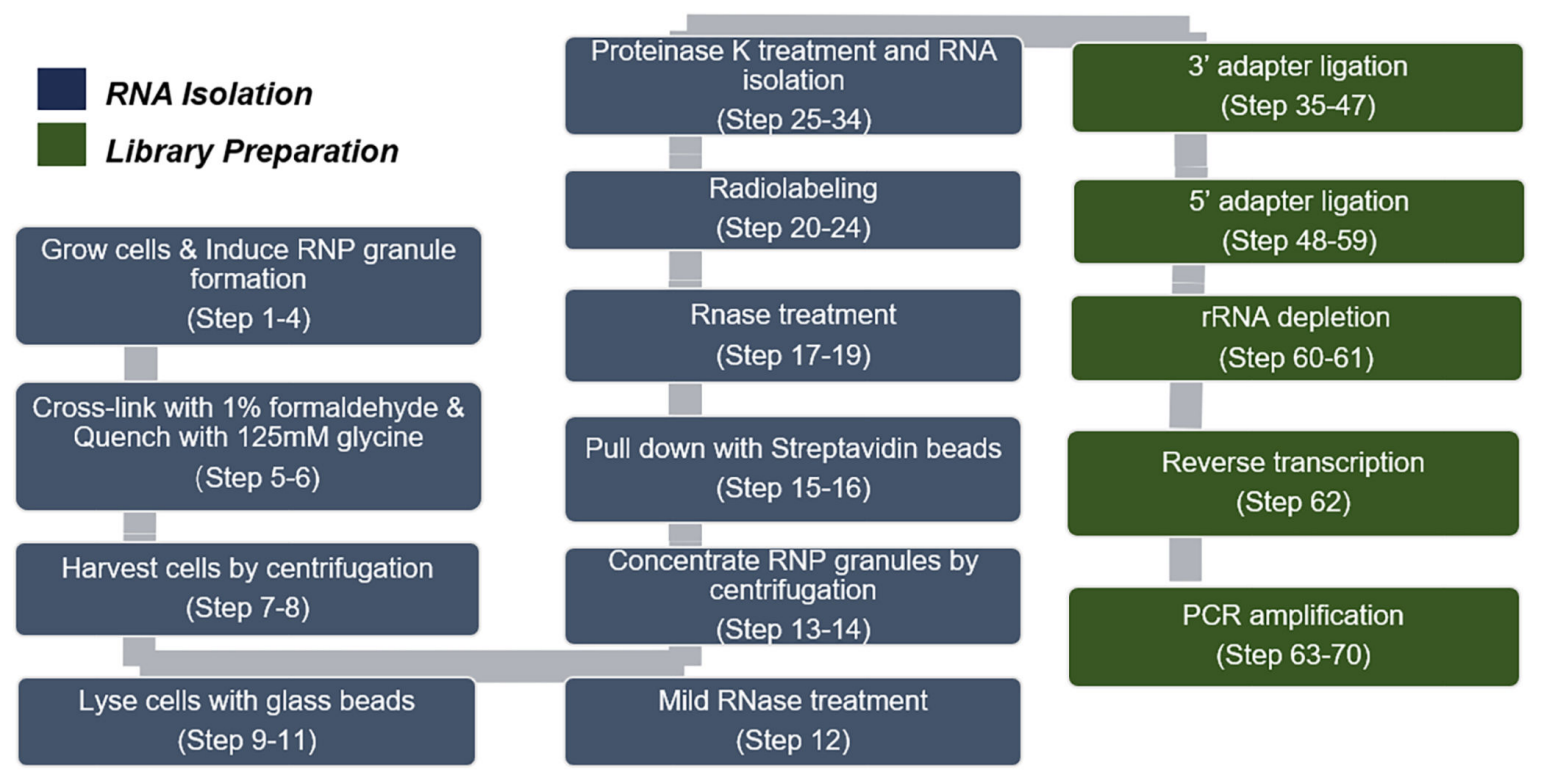
Flowchart and summary of our approach

The major steps of this protocol are depicted in Figure 1.

1. Streak corresponding yeast cells from a frozen stock by using a sterile tip on a YPD agar plate and incubate at 30 °C for about 48 h or until individual colonies grow.
2. Grow an overnight pre-culture by inoculating a single yeast colony from a YPD plate into 50 ml YPD liquid medium in an Erlenmeyer flask. Incubate at 200 rpm, 30 °C.
3. Next morning dilute overnight pre-culture into 1.5 L fresh YPD medium in a 2,800 ml polycarbonate culture flask with a final OD_600_ 0.05-0.1. Continue incubation at 200 rpm, 30 °C until OD_600_ reaches 0.5-0.8. Approximately 20-30 L culture is required per strain or treatment for low abundant RNPs such as P-bodies.
4. If necessary, apply corresponding treatment to yeast culture.
5. Crosslink cells with 37% formaldehyde at a final concentration of 1% in a fume hood and incubate at room temperature for 2 min with gentle agitation on an orbital shaker. Process 3-4 flasks each time.
6. Add glycine to a final concentration of 125 mM to quench the formaldehyde and incubate at room temperature for 5 min with gentle agitation at room temperature.
7. Harvest the yeast cells first by centrifugation at 3,000 *x g* for 5 min in centrifuge bottles at 4 °C and discard the supernatant. Resuspend the cell pellet in approximately 100 ml pre-cooled ddH_2_O, pool the suspension by centrifugation again at 3,000 *x g* for 1 min in 50 ml Falcon tubes at 4 °C and discard the supernatant. Weigh the cell pellet. Flash Freeze cell pellet in liquid nitrogen and store at -80 °C. Approximately 40 g cell pellet is required per strain or treatment for low abundant RNPs such as P-bodies.
8. Resuspend the cell pellet in prechilled RIPA buffer (2 ml buffer per gram of cell pellet).
9. Transfer the cell resuspension to 2 ml screw cap tubes, approximately 1.2 ml per tube containing 200 μl (150-212 μm) and 200 μl (450-600 μm) acid-washed glass beads. Approximately 70-90 tubes are required.
10. Lyse cells by using a Fastprep^®^ kept at 4 °C at a speed of 6.5 M/sec for 3-5 cycles of 45 sec each with 5 min ice incubations in between each lysis step. Assess the lysis under a microscope.
11. Remove large cell debris by centrifugation at low speed, 1,300 *x g* for 5 min at 4 °C. Transfer the supernatant into clean 2 ml microcentrifuge tubes. Pool two to three tubes from last step into one clean 2 ml microcentrifuge tube. Approximately 30-40 tubes are required.
12. Treat the supernatant with RNase T1 at a final concentration of 50 U/ml at 22 °C for 15 min with end over end rotation followed by 5 min on ice.
13. Pellet the membrane fraction by centrifugation at 20,000 *x g* for 10 min at 4 °C and discard the supernatant. Wash the pellet once with 1 ml RIPA buffer per tube by centrifugation at 20,000 *x g* for 10 min at 4 °C and discard the supernatant.
14. Pre-wash streptavidin beads in a 15 ml Flacon tube, once with 3-4 bed vol. nuclease-free water and once with 3-4 bed vol. incubation buffer followed by centrifugation at 800 *x g* at room temperature. Discard supernatant. Resuspend the beads with incubation buffer in a 1.5 ml or 2 ml tube to make 50% slurry.
15. Resuspend the pellets in 1 ml incubation buffer per 5-8 tubes (from Step 14) and incubate with 50 μl pre-washed streptavidin agarose beads (bed volume) per 1 ml incubation buffer in a 15 ml Falcon tube at room temperature with end over end rotation overnight. Approximately 250-400 μl streptavidin agarose beads (bed volume) is used.
16. Wash the beads 3-4 times each with 10 ml wash buffer followed by centrifugation at 800 *x g* at room temperature. Discard supernatant. During the last wash, transfer the beads into a 1.5 ml protein low LoBind tube, centrifuge at 800 *x g* at room temperature and discard supernatant.
17. Wash the beads 3 times each with 1 ml PNK buffer (without DTT) by centrifugation at 800 *x g* at 4 °C. Discard supernatant.
18. Resuspend the beads with one bed volume PNK buffer (without DTT) and add RNase T1 to a final concentration of 100 U/μl. Incubate the reaction at 22 °C for 15 min with occasional mixing. After RNase treatment, place reaction on ice for 5 min.
19. Wash the beads 5 times each with 1 ml PNK buffer (without DTT) by centrifugation at 800 *x g* at 4 °C. Discard supernatant.
20. Resuspend the beads with one bed volume NEBuffer™ 3 containing CIP at a final concentration of 0.5 U/μl. Incubate the reaction at 37 °C for 10 min with shaking at 800 rpm.
21. Wash the beads twice each with 1 ml PNK buffer (without DTT) and twice with 1 ml PNK buffer by centrifugation at 800 *x g* at 4 °C.
22. Resuspend the beads with one bed volume PNK buffer, add T4 PNK and γ-^32^P-ATP to a final concentration of 1 U/μl and 0.5 μCi/μl, respectively. Incubate the reaction at 37 °C for 30 min with shaking at 800 rpm. Set up the same reaction with 50-100 ng 19 nt ssRNA size marker in a final volume of 20 μl (All the following steps are done with the marker sample as control).
23. Add ATP into the reaction to a final concentration of 100 μM and continue to incubate for 5 min at 37 °C.
24. Wash the beads 3 times each with 1 ml PNK buffer (without DTT) and twice with 1 ml PNK buffer by centrifugation at 800 *x g* at 4 °C. Discard supernatant.
25. Wash once with one bed volume of 2x proteinase K buffer. Discard supernatant.
26. Resuspend the beads with one bed volume of 2x proteinase K buffer and add proteinase K to a final concentration of 1.2 mg/ml. Incubate the reaction at 55 °C for 30 min with shaking at 800 rpm.
27. Add one bed volume of PCI, vortex for 10 sec and spin down for 10 min at 20,000 *x g* at room temperature.
28. Transfer the aqueous phase to a 1.5 ml DNA LoBind tube.
29. Add 200 μl chloroform per tube, vortex for 10 sec and spin down for 10 min at 20,000 *x g* at room temperature.
30. Transfer the aqueous phase to a new 1.5 ml DNA LoBind tube. Heat sample at 65 °C for at least 2 h to reverse cross-linking.
31. Add NaCl to a final concentration of 0.4 M, 1 μl GlycoBlue™ and 2-3 vol. 100% Ethanol.
32. Incubate sample for at least 30 min at -80 °C for precipitation.
33. Spin down for 30 min at 20,000 *x g* at 4 °C. Discard the supernatant.
34. Wash the pellet with 200 μl of 70% ethanol by centrifugation at 20,000 *x g* at 4 °C. Discard the supernatant. Air dry the pellet.
35. Resuspend the pellet in 10 μl nuclease-free water and add 1 μl 3’ adapter (50 μM). Denature at 95 °C for 30 sec and immediately place the tube on ice for at least 3 min.
36. Assemble the 20 μl reaction by adding:

**Table:** 

50% (v/v) DMSO	6 μl
10x T4 RNA ligase buffer	2 μl
T4 RNA ligase 2, truncated	1 μl
37. Incubate on ice overnight (at least 12 h) in a cold room.
38. Pre-run gel at 250 V for at least 30 min and wash the wells extensively with 1x TBE.
39. Add 1 vol. of 2x RNA loading dye and run sample on 15% urea polyacrylamide gel with 1x TBE. Load ligated and non-ligated 19 nt size markers on the same gel.
40. Run gel at 200-250 V for 2-3 h or until the dye front (bromophenol blue) reaches 2 cm above the bottom of the gel.
41. Wrap the gel with Saran wrap and place phosphor screen on top of the gel. Scan screen at various time points (*e.g.,* 5 min-1 h) using a phosphorimager. After use, blank phosphor screen on a light source for 15-20 min.
42. Excise the gel piece around and above 3’ ligated 19nt size marker and place it into a clean 2 ml microcentrifuge tube. To cut, print a 1:1 size copy of the phosphoimager scan. Put the saran wrapped gel on top and cut at indicated sizes. If the gel piece cannot fit into one 2 ml tube, additional tube(s) is required (Figure 2).
43. Elute the RNA by incubating the gel piece with 0.4 M NaCl solution (1 ml per cm^2^ gel piece) supplemented with RNase inhibitor to a final concentration of 0.1 U/μl. Incubate for 15-20 h at 4 °C with rotation/shaking.
44. Transfer the liquid to new 1.5 ml DNA LoBind tubes, 300-400 μl per tube. Add 1 μl GlycoBlue™ and 2-3 vol. 100% Ethanol per tube.
45. Incubate sample for at least 30 min at -80 °C for precipitation.
46. Spin down for 30 min at 20,000 *x g* at 4 °C. Discard the supernatant.
47. Wash the pellet with 200 μl of 70% ethanol by centrifugation at 20,000 *x g* at 4 °C. Discard the supernatant. Air dry the pellet.
48. Resuspend the pellet in 9 μl nuclease-free water and add 1 μl 5’ adapter (50 μM). Denature at 95 °C for 30 sec and immediately place the tube on ice for at least 3 min.
49. Assemble the 20 μl reaction by adding:

**Table:** 

50% (v/v) DMSO	6 μl
10x reaction buffer (supplied)	2 μl
T4 RNA ligase 2	2 μl
Incubate at 37 °C for 1 h
50. Add 1 vol. of 2x RNA loading dye and run sample on 12% urea polyacrylamide gel with 1x TBE. Load ligated and non-ligated 19 nt size markers on the same gel.
51. Pre-run gel at 250 V for at least 30 min and wash the wells extensively with 1x TBE. Run gel at 200-250 V for 1.5-2 h or until the dye front (bromophenol blue) reaches 2 cm above the bottom of the gel.
52. Wrap the gel with Saran wrap and place phosphor screen on top of the gel. Scan screen at various time points (*e.g.,* 10 min-2 h) using a phosphorimager. After use, blank phosphor screen on a light source for 15-20 min.
53. Excise the gel piece above 3’ ligated 19 nt size marker and place it into a 2 ml microcentrifuge tube. If the gel piece cannot fit into one 2 ml tube, additional tube(s) is required.
54. Elute the RNA by incubating the gel piece with 0.4 M NaCl solution (1 ml per cm^2^ gel piece) supplemented with RNase inhibitor to a final concentration of 0.1 U/μl. Incubate 15-20 h at 4 °C with rotation/shaking.
55. Transfer the liquid to new 1.5 ml DNA LoBind tubes, 300-400 μl per tube. Add 1 μl GlycoBlue™ and 2-3 vol. 100% Ethanol per tube.
56. Incubate sample for at least 30 min at -80 °C for precipitation.
57. Spin down for 30 min at 20,000 *x g* at 4 °C. Discard the supernatant.
58. Wash the pellet with 200 μl of 70% ethanol by centrifugation at 20,000 *x g* at 4 °C. Discard the supernatant. Air dry the pellet.
59. Resuspend RNA pellet in 15 μl of nuclease-free water.
60. Deplete the rRNA by using yeast RiboMinus™ isolation kit according to the manufacturer’s instructions.
61. Elute RNA with 10-15 μl nuclease-free water into a 1.5 ml DNA LoBind tube.
62. Perform the reverse transcription (cDNA synthesis) with SuperScript™ III reverse transcriptase kit and RA3_RT primer according to the instruction manual.
63. To define the final PCR cycle number with optimum ratio between libraries and empty adapter-adapter species, perform a pilot PCR before the final PCR amplification. Assemble the reaction in a 1.5 ml DNA LoBind tube by adding:

**Table:** 

10x Taq DNA polymerase buffer (supplied with enzyme)	10 μl
dNTPs 10 mM	2 μl
PCR forward primer (RP1) 100 μM	0.5-1 μl
Indexing Primer (RPI1) 100 μM	0.5-1 μl
cDNA (direct after RT)	4 μl
Nuclease-free water	to total vol. 100 μl
64. Divide the reaction mix into 5-10 clean PCR tubes, each with 10-20 μl. Set up the PCR program as following:

**Table:** 

95 °C	1 min		
95 °C	30 sec	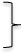	x cycles
55 °C	30 sec		
72 °C	25 sec		
72 °C	2 min
  Take out one sample each time at indicated cycle numbers (*e.g.,* 10, 12, 14, 16, 18, 20, 22, 24…) and run on 2.5% agarose gel supplemented with RedSafe™ dye (1:50,000) at 180 V for 0.5-1 h. Load low Range DNA ladder on the outermost lane on the gel.
65. Visualize the DNA with a gel imager and define the optimum cycle number (desired PCR product is clearly visible while unwanted 5’-adapter-3’-adapter is not overamplified) (Figure 3).
66. Run final PCR with defined cycle number (same PCR program). Assemble the reaction in a PCR tube by adding:

**Table:** 

10x Taq DNA polymerase buffer (supplied with enzyme)	20 μl
dNTPs 10 mM	4 μl
PCR forward primer (RP1) 100 μM	1-2 μl
Indexing Primer (RPI1-4) 100 μM	1-2 μl
cDNA (direct after RT)	8 μl
Nuclease-free water	to total vol. 200 μl
67. Run PCR products on 2.5% agarose gel supplemented with RedSafe™ dye (1:50,000) at 180 V for 1 h. Load low Range DNA ladder on the outermost lane on the gel.
68. Visualize the DNA with a gel imager and cut out the gel piece corresponding to your library.
69. Use NucleoSpin^®^ gel purification kit to isolate library from the gel according to the manufacturer’s instruction manual. Elute with 20 μl nuclease-free water into a 1.5 ml DNA LoBind tube.
70. Assess the library with Bioanalyzer. Proceed to sequencing.

## Notes

In this protocol, Fastprep^®^ was used to lyse the yeast cells. To improve the lysis efficiency, a Freezermill could be used. The starting material (amount of cells) may vary according to the abundancy of target RNPs. A centrifugation step is included in our protocol (Step 12) to separate cytosolic from membrane fractions since our target mRNP granules (PBs) have been shown to be associated with ER membrane. Depending on the expected library size, different ssRNA size markers and/or RNA ladder could be used when running a polyacrylamide gel.

## Recipes

1. Yeast Extract-Peptone-Dextrose (YPD) agar
  1% (w/v) Yeast extract
  2% (w/v) Peptone
  2% (w/v) Dextrose
  2% Agar
2. Yeast Extract-Peptone-Dextrose (YPD) medium
  1% (w/v) Yeast extract
  2% (w/v) Peptone
  2% (w/v) Dextrose
3. RIPA buffer
  50 mM Tris-HCl (pH 8.0)
  150 mM NaCl
  1% (v/v) NP-40
  0.5% (w/v) Sodium deoxycholate
  0.1% (w/v) SDS
  Add proteinase inhibitor cocktail before use, one tablet per 50 ml buffer
4. Incubation buffer
  50 mM sodium phosphate buffer (NaPi, pH 8.0)
  300 mM NaCl
  6 M GuHCl
  0.5% (v/v) TWEEN^®^ 20
5. Wash buffer
  50 mM sodium phosphate buffer (NaPi, pH 8.0)
  300 mM NaCl
  6M GuHCl
  0.5% (v/v) TWEEN^®^ 20
  0.5% (v/v) NP-40
6. PNK buffer
  50 mM Tris-HCl (pH 7.5)
  50 mM NaCl
  10 mM MgCl_2_
  5 mM DTT
7. 2x proteinase K buffer
  100 mM Tris-HCl (pH 7.5)
  200 mM NaCl
  2 mM EDTA
  1% (w/v) SDS
8. Urea polyacrylamide gel
  15% or 12% (w/v) Acrylamide/Bis-acrylamide
  8 M Urea
  Add 1x TBE buffer to desired volume
  Further add 40 μl 10% (w/v) APS and 4 μl TEMED per 10 ml
9. Tris-HCl buffer (1M)
  121.14 g Tris base
  Adjust the pH by adding HCl at RT

## Figures and Tables

**Figure 2:**
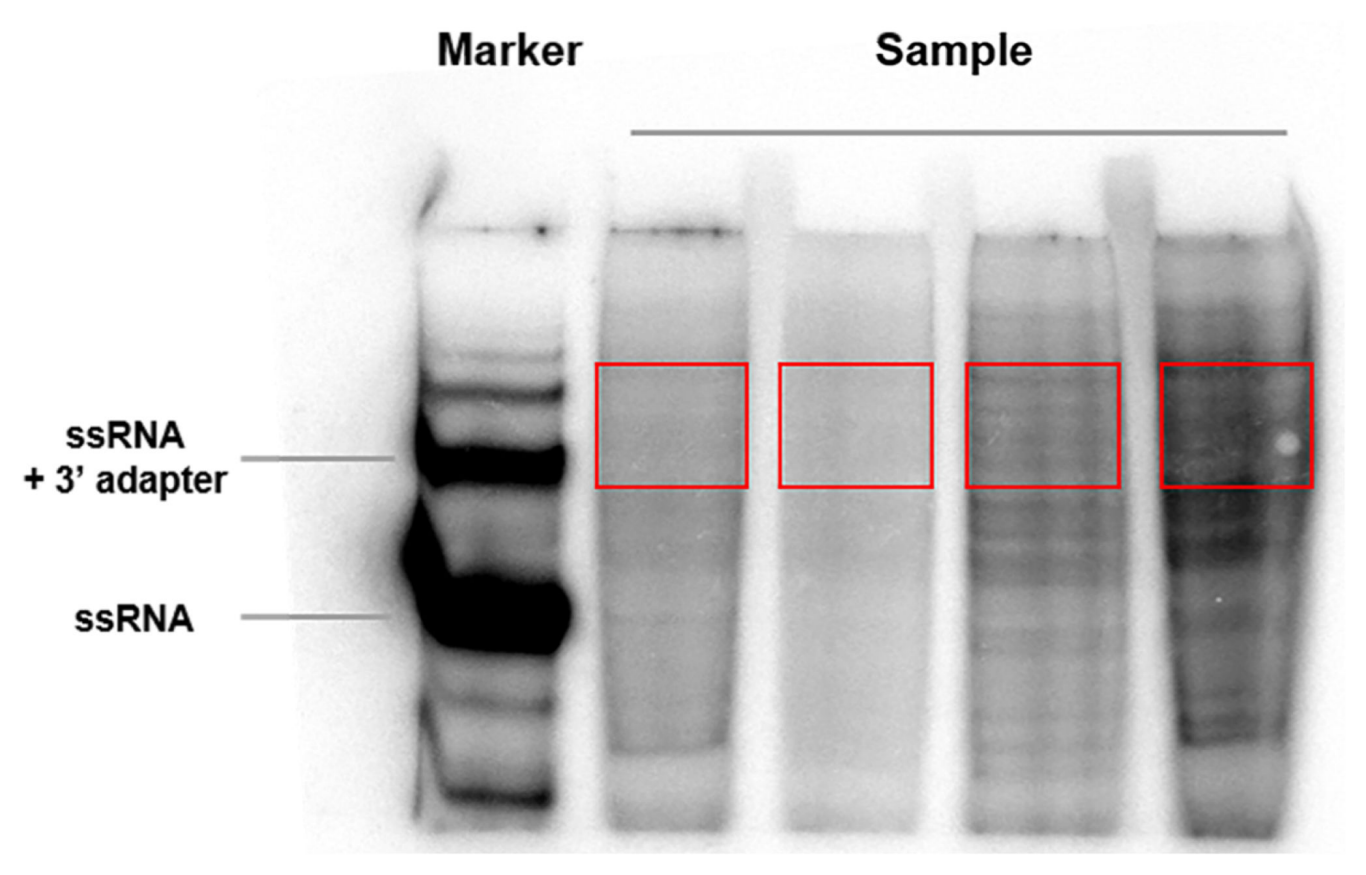
Example gel picture after 3’ adapter ligation. The red rectangle indicates the gel region to be excised.

**Figure 3:**
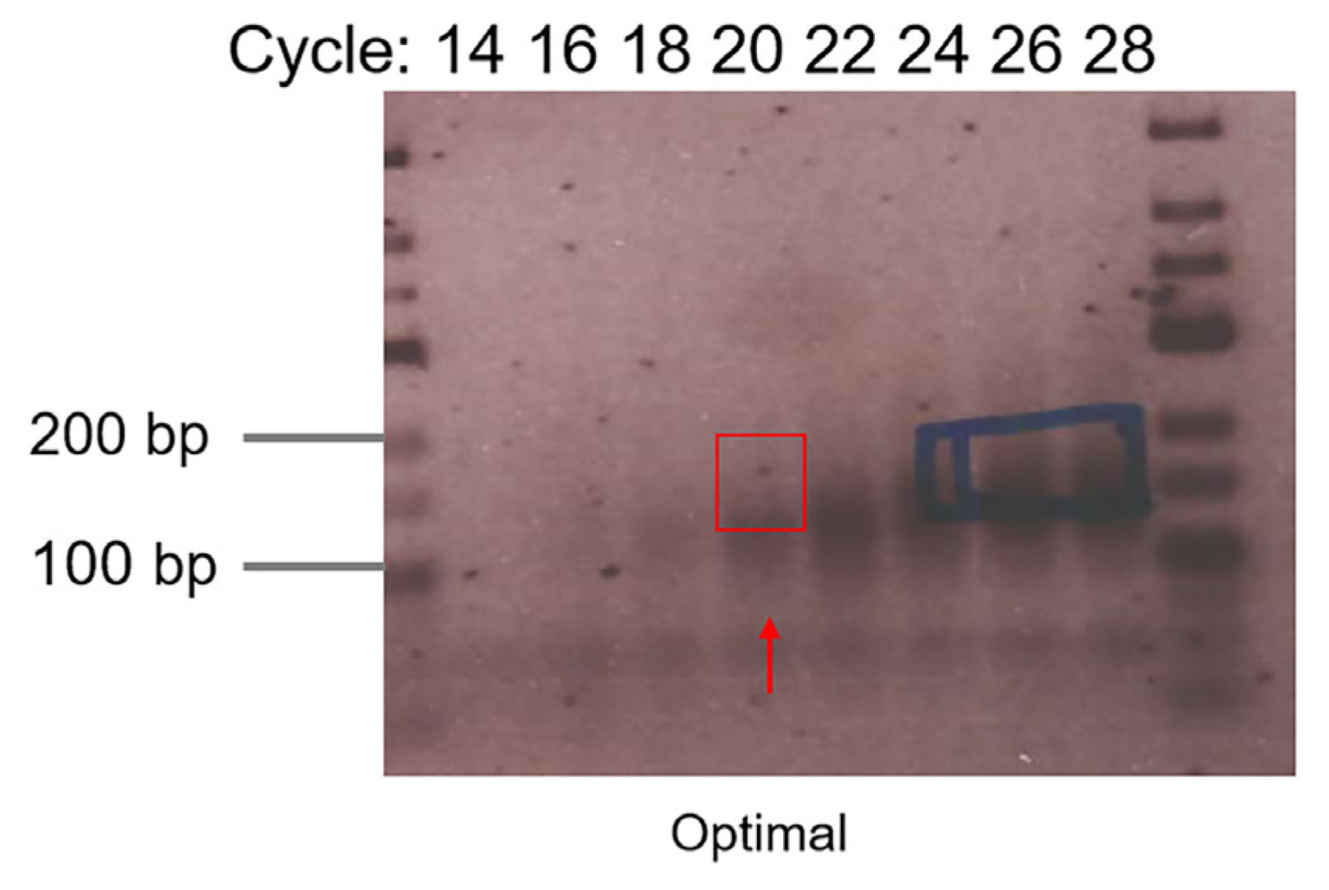
Example gel picture of test PCR. The red arrow indicates the optimal cycle number. The red rectangle indicates the gel region to be excised. The blue rectangle shows overamplifications which increase 5’-adapter-3’-adapter species.
